# Integrated Strain–Flow Analysis for Early Assessment of Right Ventricular Dysfunction in Pulmonary Arterial Hypertension

**DOI:** 10.1007/s10439-026-04153-2

**Published:** 2026-05-01

**Authors:** Mohammad Saber Hashemi, Ahmad Falahatpisheh, Yasaman Farsiani, Yuri Matusov, Siddharth Singh, Kambiz Ghafourian, Gianni Pedrizzetti, Arash Kheradvar

**Affiliations:** 1 Department of Biomedical Engineering, University of California, Irvine, CA, USA; 2 Division of Pulmonary & Critical Care Medicine, Cedars Sinai Medical Center, Los Angeles, CA, USA; 3 Cedars Sinai Medical Center, Los Angeles, CA, USA; 4 Department of Engineering and Architecture, University of Trieste, Trieste, Italy; 5 Department of Radiological Sciences, University of California, 2420 Engineering Hall, UC Irvine, Irvine, CA 92697, USA

**Keywords:** Right ventricular function, Pulmonary arterial hypertension, 3D echocardiography, Volumetric echocardiographic particle image velocimetry

## Abstract

**Purpose:**

Early detection of right ventricular (RV) dysfunction is essential in pulmonary arterial hypertension (PAH) but remains challenging using conventional echocardiography. This study investigates the feasibility of a noninvasive, physics-based framework using three-dimensional (3D) echocardiography that integrates myocardial strain and volumetric flow analysis to characterize RV mechanical performance across stages of PAH.

**Methods:**

A prospective pilot study (N=15) enrolled healthy controls, PAH patients with preserved RV size, and PAH patients with RV dysfunction. Deformation was evaluated by principal strain analysis and by conventional (longitudinal, circumferential) components. Hemodynamic metrics included hemodynamic forces and energetic properties that were derived using a physics-informed volumetric echocardiographic particle image velocimetry (V-Echo-PIV) method applied to contrast-enhanced acquisitions.

**Results:**

Deformation analysis revealed that longitudinal strain was significantly reduced even in PAH patients with preserved RV dimensions, while second principal (secondary) strain showed a distinctive sign reversal, indicating a paradoxical systolic lengthening, early in the disease. The analysis of hemodynamic forces showed a marked reduction in systolic propulsion across all PAH stages. In contrast, energetic abnormalities were predominantly observed at later stage of the disease.

**Conclusions:**

The integration of 3D myocardial strain with fluid dynamics provides a comprehensive physiological assessment of RV remodeling. While strain and systolic propulsion appear as sensitive markers for early dysfunction, diastolic energetics may support disease staging. This noninvasive framework shows promise for early detection and longitudinal monitoring of PAH patients.

## Introduction

Pulmonary arterial hypertension (PAH) is a progressive cardiopulmonary disorder characterized by elevated pulmonary arterial pressure and increased pulmonary vascular resistance. Chronic pressure overload leads to maladaptive right ventricular (RV) remodeling, subsequently leading to dilation, dysfunction, and ultimately RV failure [1–3]. In the U.S., an estimated 500 to 1000 new PAH cases are diagnosed each year, with no clear racial or ethnic predisposition [4]. Although relatively uncommon, PAH carries a disproportionately high burden of morbidity and mortality, partly due to delayed diagnosis stemming from mild and nonspecific early symptoms [5, 6]. PAH guidelines highlight the urgent need for early detection of clinically meaningful RV dysfunction that can guide timely treatment escalation [7–9]. RV function is a key predictor of prognosis and mortality [1], correlating closely with clinical severity and quality of life [10]. Therefore, the development of robust quantitative tools to assess RV performance and track disease progression is critical for anticipating the onset of RV dysfunction and enabling timely initiation of PAH-specific therapies to delay progression toward RV failure.

Right heart catheterization (RHC) is the gold standard for the diagnosis and monitoring of PAH. Cardiac imaging (particularly echocardiography due to its widespread availability) provides numerous insights in into RV structure and function: echocardiographic markers of RV dysfunction—such as RV size and ejection fraction [11–15], tricuspid annular velocity [16–18], tricuspid annular plane systolic excursion (TAPSE) [19], RV index of myocardial performance (RIMP) [19], tricuspid regurgitation [11, 20, 21], and Doppler-derived dP/dt [22]—are well established and widely used to characterize structural and functional changes. Although these imaging parameters inform RV function and its progression, they provide limited insight into hemodynamic load and lack specificity for PAH and therefore remain complementary rather than diagnostic compared to invasive measurements from right heart catheterization (RHC).

Progression of PAH is typically marked by RV dilation. This volumetric increase is preceded by an earlier, subclinical stage of RV dysfunction, during which the chamber size remains normal. Because this early dysfunction is not accompanied by gross anatomical changes, it is difficult to detect using standard imaging. However, early recognition of these subtle functional changes could enable earlier therapeutic escalation, potentially slowing disease progression and improving clinical outcomes in PAH patients [23, 24].

An integrated biomechanical assessment of RV function—incorporating myocardial deformation and intraventricular blood flow characteristics—has the potential to provide complementary, noninvasive insight into RV hemodynamic efficiency, improve physiologic interpretation, and enable earlier detection of PAH-related dysfunction. The present study applies a physics-based framework to comprehensively characterize RV mechanical function using three-dimensional (3D) echocardiography. RV myocardial deformation and intraventricular pressure gradients are derived directly from segmented 3D geometries, while energetic properties of intraventricular flow are estimated using a novel volumetric echocardiographic particle image velocimetry (V-Echo-PIV) method applied to contrastenhanced echocardiography. This feasibility study evaluates the proposed approach in a cohort spanning a range of RV dysfunction to assess its potential for noninvasive support of PAH-related RV assessment.

## Methods

### Clinical Study Design and Participant Details

This prospective, observational feasibility study was conducted at a single tertiary pulmonary hypertension center with a high-volume outpatient clinic. The study was approved by the institutional review boards of Cedars-Sinai Medical Center (STUDY00001683), and the University of California, Irvine (Protocol #20216931), and written informed consent was obtained from all participants.

Consecutive adults referred for clinical evaluation of suspected or established PAH were prospectively enrolled and underwent standard transthoracic echocardiography with additional 3D acquisitions for RV mechanical assessment. Participants were categorized into three cohorts based on hemodynamic status and RV function: PAH (normal RV function), PAH+RVD (PAH with RV dysfunction), and healthy controls, based on the clinical criteria described below.

#### PAH with normal RV function cohort (PAH):

Patients met guideline-based diagnostic criteria for PAH, defined as mean pulmonary arterial pressure (mPAP) ≥ 25 mmHg at rest, pulmonary artery wedge pressure (PAWP) ≤ 15 mmHg, and pulmonary vascular resistance (PVR) > 3 Wood units. In addition, they were required to have normal RV function on conventional echocardiography.

#### PAH with RV dysfunction cohort (PAH+RVD):

Patients met the same hemodynamic criteria for PAH but demonstrated RV dysfunction on echocardiography, defined according to ASE guideline thresholds: TAPSE < 16 mm; RIMP > 0.40 by pulsed Doppler or > 0.55 by tissue Doppler; and/or tricuspid annular systolic velocity (S’) < 10 cm/s.

#### Healthy control cohort (Control):

Control subjects were recruited from the community through public advertisement. Eligible individuals were ≥ 18 years of age, had no history or clinical suspicion of cardiovascular or pulmonary disease, and met the same imaging quality requirements as the PAH cohorts.

Across all cohorts, participants were required to be in sinus rhythm and to have adequate acoustic windows for 3D RV imaging. Exclusion criteria included prior congenital or acquired right-heart surgery, significant left-or right-sided valvular disease, intracardiac shunts, or arrhythmias that impaired image acquisition.

All echocardiographic imaging was performed by experienced sonographers following established clinical guidelines [25] and study protocols to ensure consistency across participants. Echocardiographic datasets were exported and stored in DICOM format for standardized post-processing and analysis.

Continuous variables are reported as mean ± SD. Comparisons between PAH and control groups were performed using the Mann–Whitney U test, while comparisons among the three independent groups were evaluated using the Kruskal–Wallis H test. Given the small sample size (N=15;n=5 per group), this study is intended as an exploratory pilot investigation focused on feasibility. Accordingly, all statistical analyses are underpowered for definitive inference, and reported p-values should be interpreted as descriptive rather than confirmatory.

### Echocardiography Acquisition Protocol

Transthoracic echocardiography (TTE) was performed using a Philips EPIQ 7 ultrasound system (Philips Health-care, Andover, MA) equipped with a 3D matrix-array transducer (X5-1) to acquire B-mode images of the RV chamber throughout the cardiac cycle. RV-focused apical four-chamber views (A4C) were obtained in full-volume mode, optimizing acquisition by minimizing sector size and depth to solely include the RV from tricuspid annulus to apex. Tissue harmonic imaging was used to reduce background noise and side-lobe artifacts. To optimize spatial resolution based on patient anatomy and acoustic window, HPen or HRes modes were selected, Frame rate+ mode was activated, and manual settings (narrow sector, shallow depth) allowed frame rates above 30 Hz. Four-beat full-volume acquisitions yielded up to 100 Hz in patients with sinus rhythm.

Additional images were acquired during infusion of a contrast agent to visualize RV flow. For this purpose, identical imaging settings were maintained, while contrast-specific settings—commonly used to enhance contrast intensity and achieve homogeneous chamber opacification—were deliberately avoided to preserve temporal resolution and improve visualization of microbubble dynamics. A custom IV mixture of Optison^™^ contrast agent and 0.9% normal saline (in a 2:30 to 2:60 [Optison:NS] ratio), followed by a 1–2 mL saline flush, was administered to enhance bubble visibility. Recording began approximately 4–6 seconds post-injection, once contrast saturation decreased and microbubble motion became visually discernible. The mechanical index—an echocardiographic parameter representing the acoustic power of the ultrasound wave, defined as the ratio of peak negative pressure to the square root of frequency—was maintained at low values (0.4–0.6) to ensure sufficient contrast enhancement and endocardial delineation while limiting microbubble disruption [26]. This approach has been previously applied in clinical studies using 2D imaging and was directly adapted to the present application [27, 28].

**Fig. 1:**
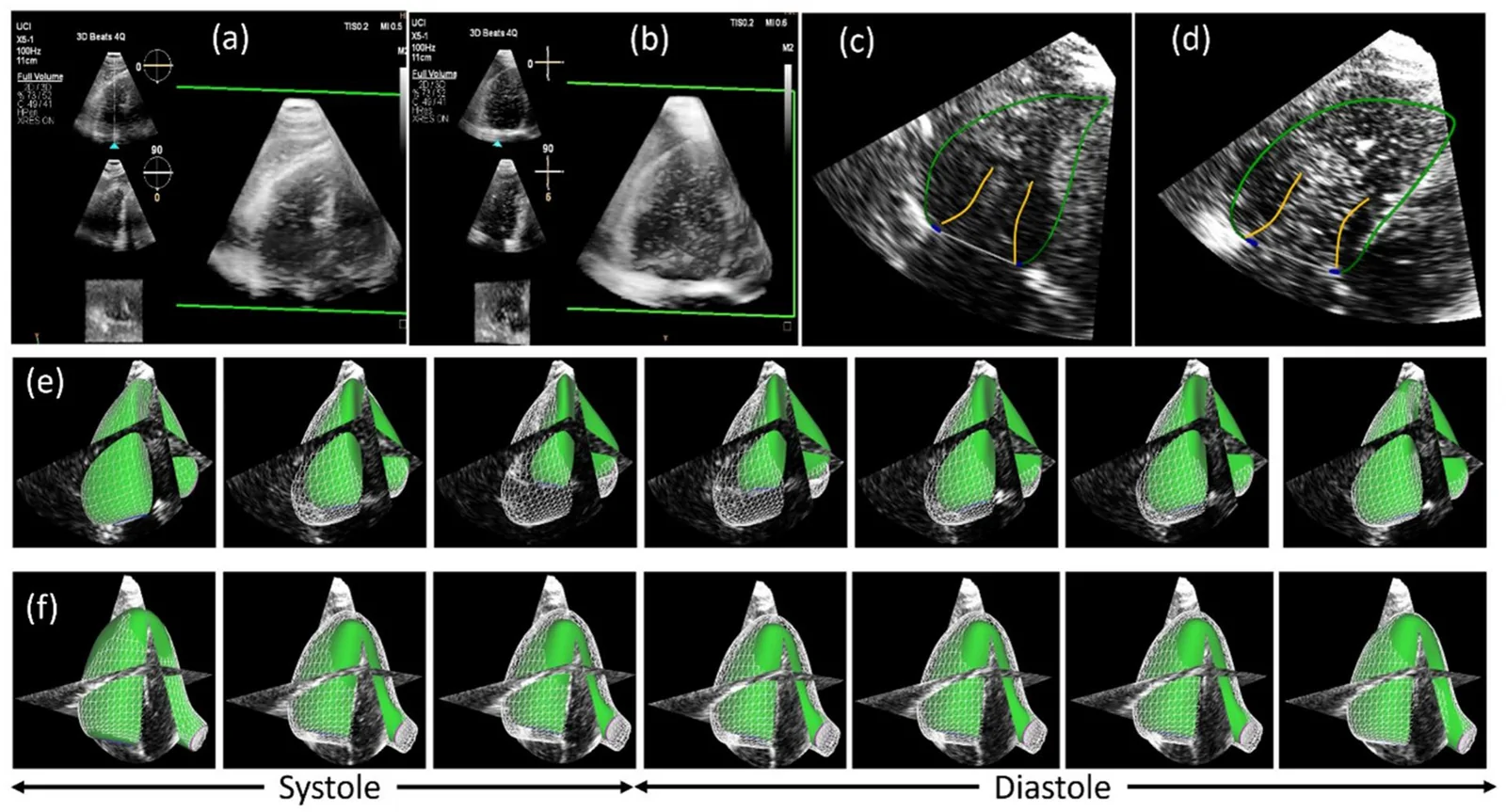
RV-focused 4D acquisition with 4-beat stitching results in 100 Hz in the presence of contrast bubbles, in a normal RV **a**, **c** versus a dilated RV **b**, **d**. RV Volume dynamics (green surface) is displayed with reference to the end-diastolic surface (white mesh) throughout a cardiac cycle in a normal RV **e** and in a dilated RV due to pulmonary hypertension **f**, as processed by the TomTec software

RV chamber segmentation was conducted using a dedicated software (4D RV-Function, TomTec Imaging Systems GmbH, Unterschleißheim, Germany). After manual placement of anatomical landmarks (apex and tricuspid annulus), the software automatically identifies RV geometry at end-diastole (ED) and end-systole (ES). The initially proposed contours are often inaccurate, requiring manual refinement, which is typically necessary. Once ED and ES contours are finalized, the software applies speckle-tracking to propagate the endocardial geometry throughout the cardiac cycle, as illustrated for a representative subject in Fig. 1. The resulting RV surface mesh was then exported as a triangulated surface for further analysis.

### RV Deformation Analysis

Multidirectional RV strain was computed using a 4D echocardiography-based approach adapted from prior work originally developed for the left ventricle (LV) by Pedrizzetti et al. [29, 30] and later extended to pediatric congenital heart disease patients [31–33]. Lagrangian 2D strain tensors were calculated for each triangle of the RV endocardial surface mesh, using the end-diastolic (ED) frame as the reference undeformed configuration [34]. A principal strain analysis [35, 36] was applied to each tensor to determine directions of maximum (principal) and minimum (secondary) strain. This tensor-based method provides direction-independent characterization and is especially appropriate for the RV’s complex geometry [32, 33]. In addition to the principal components, conventional longitudinal (base-to-apex) and circumferential strain components were also derived from the same tensor for comparison. In summary, global strain time profiles (global principal strain, *GPS*; global secondary strain, *GSS*; global longitudinal strain, *GLS*; global circumferential strain, *GCS*) were computed by averaging strain values over the entire RV endocardial surface using an area-weighted sum. A single systolic strain index was extracted at ES to represent peak contraction for each component.

### Hemodynamic Forces

The intraventricular pressure gradient is the primary force driving blood flow within and through the ventricle, slightly opposed by viscous friction. When integrated over the entire RV chamber, it reflects the net force exerted by the myocardial wall to eject blood into the pulmonary artery during systole or the reciprocal interaction between the blood and endocardium during diastolic filling [37, 38]. The total hemodynamic force (HDF) is thus computed by the rate of change of momentum in the RV volume, V(t)

(1) $$F\left(t\right)=\rho \int_{V\left(t\right)}\left[\frac{\partial \text{u}}{\partial t}+\left(\text{u}\cdot \nabla \right)\text{u}\right]dV≃\int_{V\left(t\right)}\nabla pdV,$$

where u(x,t) is the fluid velocity vector field. The force vector (1) includes contributions from both the pressure gradient and viscous stresses; however, the latter is significant only near boundaries and contributes minimally to the global balance. Therefore, the force can be approximated by the volume integral of the pressure gradient, ∇p, and the hemodynamic force (normalized by volume) can be used interchangeably with the intraventricular pressure gradient. The integral of momentum in (1) can be recast as a surface integral [37, 38]

(2) $$F\left(t\right)=\rho \int_{S\left(t\right)}\left[\text{x}\left(\frac{\partial \text{u}}{\partial t}\cdot \text{n}\right)+\text{u}\left(\text{u}\cdot \text{n}\right)\right]dS,$$

over the boundary S(t) of the RV volume, and n is the local normal unit vector. HDHs are here computed by the integral (2), that becomes a sum over all triangles of the RV surface mesh, where the velocity at the endocardium is given by time differentiation of the moving geometry, and the average velocity across the open valve is obtained by continuity.

To enable comparison across subjects, the HDF was normalized with the RV volume, thus representing the average pressure gradient; it is then common practice to divide by blood density and gravitational acceleration, and express it as a percentage of gravity. The longitudinal component of the HDF—aligned from apex to base—constitutes the dominant directional contribution to RV flow. Representative parameters were calculated by average measures of the longitudinal HDF over specific phases of the cardiac cycle following the protocol described before [39]. Two key metrics were extracted: the amplitude, computed as the root mean square (RMS) of the longitudinal HDF during systole and diastole separately, *HDF-Asys* and *HDF-Adia*, and the systolic impulse, *HDF-Isys*, calculated as the time integral of positive HDF values during the propulsive systolic phase, representing the net driving effort exerted by the right ventricle.

### RV Flow Velocity Estimated by V-Echo-PIV

Contrast-enhanced echocardiographic imaging captures the motion of microbubbles within the RV chamber, which displace between successive frames according to the local flow velocity [40]. The present V-Echo-PIV method [41, 42] starts with an estimation of the instantaneous three-dimensional velocity field between two consecutive frames at each voxel using the hierarchical Lucas–Kanade optical flow algorithm. This procedure begins with a coarse volumetric interrogation window of 32^3^ voxels, which is then iteratively reduced to refining the local velocity estimates with increasing spatial resolution.

Velocity fields derived purely from image-based methods inherently provide an upper bound on the measurable velocity amplitude, limited by the temporal resolution of the acquisition. Consequently, the absolute magnitude of the velocity tends to be underestimated, although the flow direction remains reliable [43]. This underestimation occurs because these methods rely on tracking coherent microbubble patterns between consecutive frames. When microbubbles travel long distances between frames, local velocity gradients and flow mixing cause decorrelation, leading to reduced tracking accuracy. As a practical guideline, the maximum reliably measurable velocity magnitude (in cm/s) is approximately 0.5 times the imaging frame rate (in Hz) [43, 44]. Therefore, for state-of-the-art clinical ultrasound systems operating at around 100 Hz, the upper limit for directly resolvable velocities is on the order of some tens of centimeters per second.

To partially overcome this limitation, we introduce a physics-informed correction that constrains the velocity field to satisfy both (1) the known motion of the RV endocardial boundary and (2) continuity equation that in turn ensures the correct flow rate across the tricuspid and the pulmonary valves [38]. Let $u^{\left(0\right)}(x,t)$ denote the initial velocity field estimated from V-Echo-PIV. By imposing the known boundary velocities, we obtain a corrected intermediate field ${\hat{u}}^{\left(0\right)}(x,t)$ consistent with RV wall motion. However, this field may not yet satisfy the incompressibility condition.

Conservation of mass is enforced through a fractionalstep correction analogous to the method used in immersed boundary computational fluid dynamics (CFD) frameworks [45, 46]. Specifically, we seek a scalar potential field ϕ(x,t) such that the new velocity,

(3) $${\text{u}}^{\left(1\right)}={\overset{ˆ}{\text{u}}}^{\left(0\right)}+\nabla \phi ,$$

satisfies the divergence-free condition $\nabla \cdot {\text{u}}^{\left(1\right)}=0$. Since this correction alters the boundary velocities, the prescribed RV wall motion is re-imposed to obtain a new corrected field ${\hat{\text{u}}}^{\left(1\right)}$, and the mass-conservation correction is repeated. Iterating this process rapidly converges on a solution that simultaneously enforces boundary conditions and mass conservation. In practice, only a few iterations are required, and the computational cost represents a minor fraction of the total V-Echo-PIV processing time. This correction effectively refines high inflow and outflow velocities, yielding a physically consistent velocity field. However, because the correction is primarily irrotational away from the boundaries, the rotational dynamics of the cavity remain largely governed by the initial V-Echo-PIV measurements. As a result, overall accuracy remains dependent on the frame rate, which cannot be significantly lower than that employed in the present study.

Although the estimated velocities may not represent instantaneous, pointwise ground-truth measurements of blood flow, the resulting field robustly captures the overall flow organization and energy distribution. Consequently, it enables quantitative assessment of global flow features that may help to distinguish normal RV hemodynamics from pathological alterations.

### Energetic and Vortex Properties Derived from the Velocity Field

The present analysis focuses on the energetic characteristics of the RV flow field, evaluated as spatially integrated quantities normalized by chamber volume, thereby allowing intersubject comparisons independent of ventricular size.

The instantaneous kinetic energy per unit volume is defined as

(4) $$\text{KE}\left(t\right)=\frac{1}{V\left(t\right)}\int_{V\left(t\right)}\frac{1}{2}|\text{u}{|}^{2}dV,$$

expressed in $\left({\text{cm}}^{2}/{\text{s}}^{2}\right)$, which quantifies the time-varying energy content of the intracavitary blood flow [47]. The rate of kinetic energy dissipation per unit volume is given by

(5) $$\varepsilon \left(t\right)=\frac{\nu }{V\left(t\right)}\int_{V\left(t\right)}\sum_{i,j}\left(\frac{\partial u_{i}}{\partial x_{j}}\frac{\partial u_{i}}{\partial x_{j}}+\frac{\partial u_{i}}{\partial x_{j}}\frac{\partial u_{j}}{\partial x_{i}}\right)dV,$$

where $\nu =0.035{\;\text{cm}}^{2}/\text{s}$ is the kinematic viscosity of blood [47]. The dissipation rate ε(t), expressed in (cm^2^/s^3^), represents the rate at which kinetic energy is converted into heat by viscous frictional effects. To characterize global vortex dynamics without relying on explicit vortex-core segmentation methods, we introduce the vortex impulse as a volumetric measure of the rotational flow momentum

(6) $$I\left(t\right)=\frac{1}{V\left(t\right)}\int_{V\left(t\right)}x\times \omega dV,$$

where ω=∇×u is the vorticity field. The vector quantity I(t), measured in (cm/s), represents the impulse associated with vortex formation [48], whose rate of change corresponds to the net force (normalized by mass) required to generate the observed rotational motion. The component, $I_{z}(t)$, orthogonal to the tricuspid valve plane, is particularly relevant for monitoring the development of the diastolic vortex during RV filling. From these time-dependent quantities, we further derive integral energetic metrics that characterize the RV flow during systole and diastole. The average kinetic energy represents the temporal average of KE(t) over each cardiac phase, providing an index of the overall energy stored in blood motion; it is computed separately during systole, ${\bar{\text{KE}}}_{\text{sys}}$, and during diastole, ${\bar{\text{KE}}}_{\text{dia}}$, to verify the presence of alteration of energetic content during the different phases of the heartbeat. The kinetic energy loss computed as the time integral of ε(t) is indicative of the cumulative energy dissipated through viscous mechanisms during systole, ${ℒ}_{\text{sys}}$ and during diastole, ${ℒ}_{\text{dia}}$, to analyze flow efficiency. Finally, the mean diastolic vortex impulse, ${ℐ}_{\text{dia}}$, corresponds to the temporal average of the vertical component of the vortex impulse, $I_{z}(t)$, during ventricular filling.

## Results

**Fig. 2:**
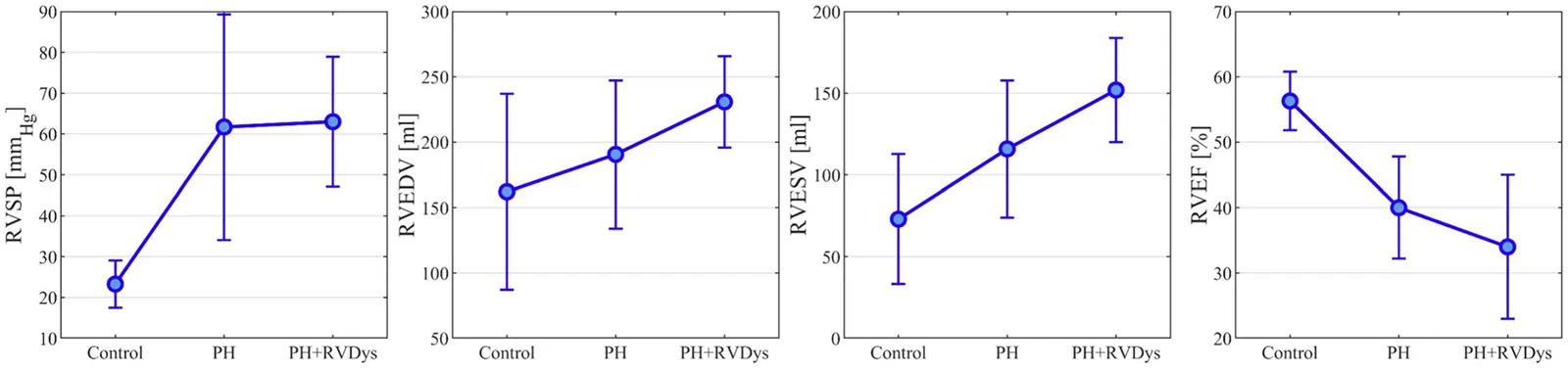
Clinical and volumetric parameters in the analyzed groups (graphic representation of data in Table 2). Abbreviations used include RVSP (right ventricular systolic pressure), RVEDV (right ventricular end-diastolic volume), RVESV (right ventricular end-systolic volume), and RVEF (right ventricular ejection fraction)

Demographic, clinical, and volumetric properties of the analyzed population are summarized in Table 1 and Fig. 2. Diagnosis and staging were performed by a board-certified pulmonologist based on comprehensive clinical assessment and invasive right heart catheterization. By study design, all patients are characterized by an elevated RV systolic pressure. Volumetric data, with control values already at the upper end of the normal range, demonstrates a clear progression of RV remodeling with increasing disease severity. Patients with PAH exhibited higher volumes than controls, approaching or exceeding normal limits, whereas PAH+RVD patients displayed overt ventricular dilation. However, RV systolic function, as assessed by ejection fraction (RVEF), is significantly reduced in all PAH patients. This pattern is consistent with conventional RV functional parameters, such as TAPSE, which are reduced across all patients, with more pronounced impairment in the presence of RV dysfunction.

As an exploratory pilot study, these findings represent reference values in the analyzed population in relation to the following evaluation of mechanical function rather than definitive group differences.

### Preliminary Verification of the V-Echo-PIV Method

A preliminary verification of the V-Echo-PIV method was conducted to assess numerical consistency, convergence behavior, and physiological plausibility. This step is intended to verify that the algorithm operates as designed and yields physically consistent results; comprehensive experimental validation against independent MRI-based flow measurements will be pursued in future work. The analysis was performed on representative subjects from each of the three study groups, thereby encompassing a spectrum of physiological and pathological conditions.

**Fig. 3:**
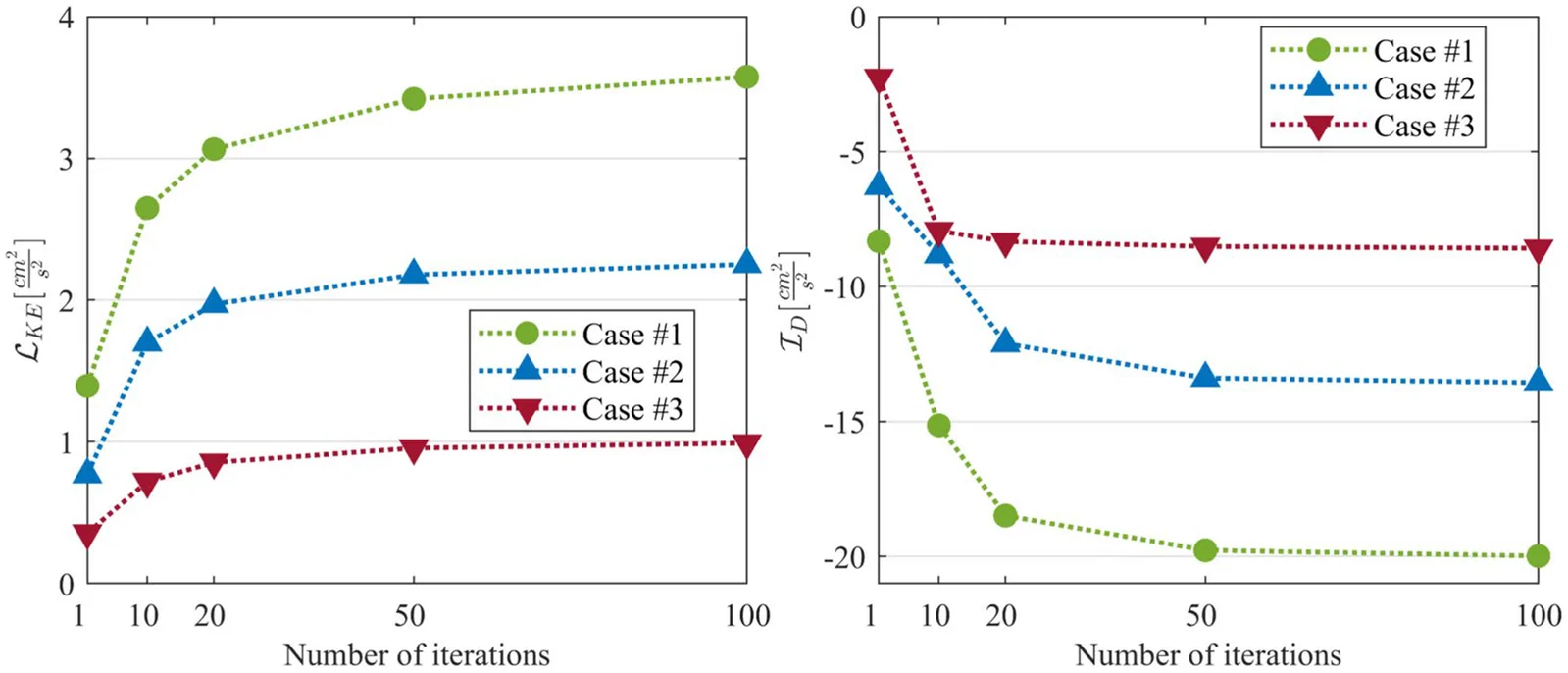
Analysis of the convergence of flow properties with increasing the number of iterations. The total loss of kinetic energy and the average diastolic impulse are reported on the left and right graphs, respectively, as a function of the number of iterations

First, the convergence behavior of the iterative correction process is illustrated in Fig. 3, which shows the iterative evolution of kinetic energy loss $\left({ℒ}_{\text{KE}}\right)$ over the cardiac cycle and the mean tricuspid vortex impulse during diastolic filling $\left({ℐ}_{D}\right)$ for a control subject (Case #1), a PAH patient (Case #2), and a PAH + RVD patient (Case #3). The results demonstrate rapid convergence—good agreement after approximately 10 iterations and excellent stability after 20 iterations. To further confirm internal consistency, the total vorticity within the $\text{RV}\left(\int_{V}\omega \text{dV}\right)$ was monitored. This integral, a theoretical invariant of the flow field, should ideally remain zero throughout the cardiac cycle. When normalized by the square root of the enstrophy $\left(\int_{V}|\omega {|}^{2}\text{dV}\right)$, the resulting dimensionless components remained on the order of 10^−4^, and the magnitude never exceeded 10^−3^ after the first iteration, confirming the numerical stability and physical coherence of the method.

**Fig. 4:**
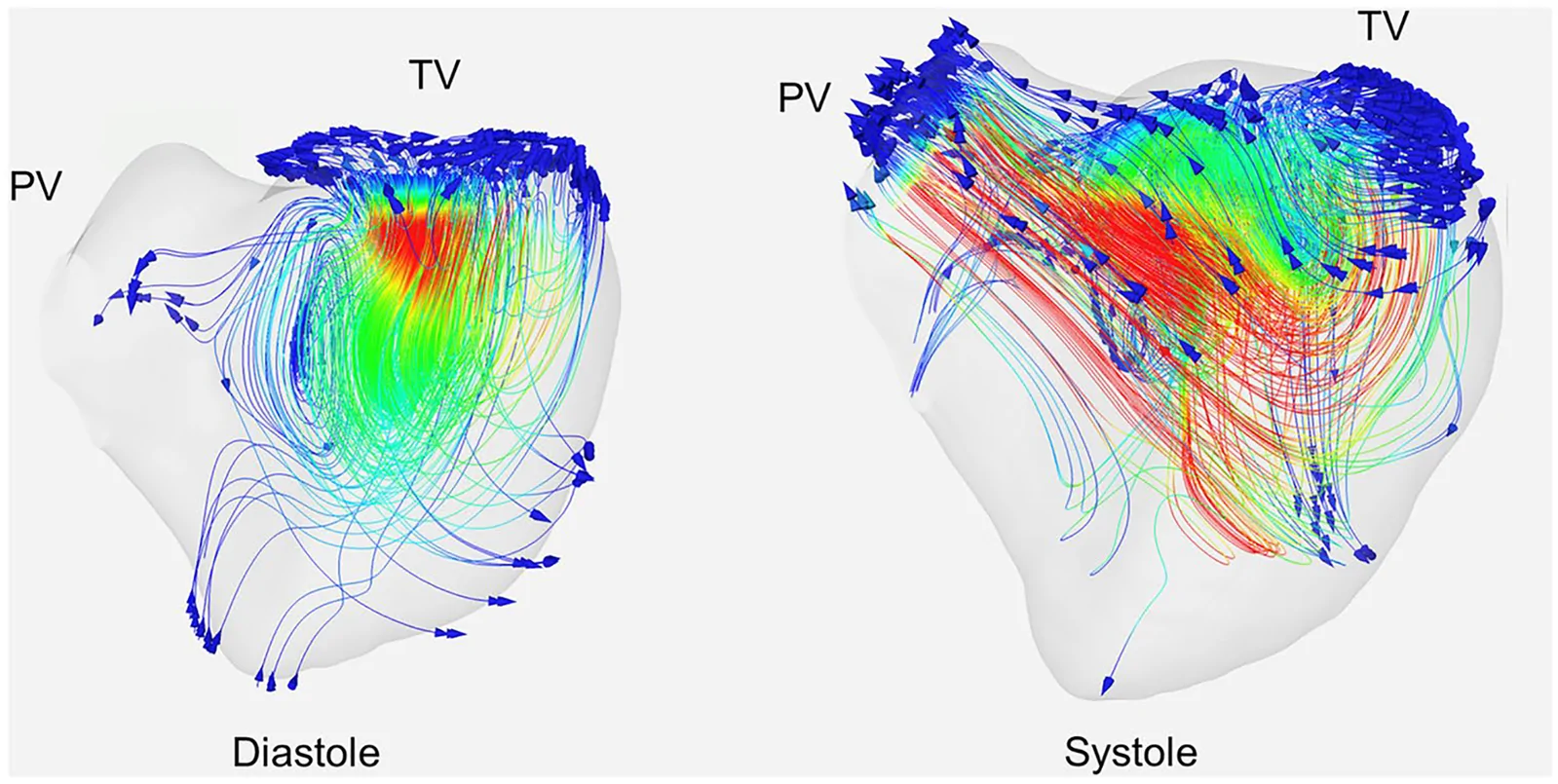
Flow visualization of the RV flow field in a normal subject (Case #1) during diastolic filling and systolic ejection (left and right pictures, respectively). Flow is visualized in terms of streamlines (emitted from a horizontal plane at the level of the tricuspid valve at diastole) and colored by the magnitude of velocity (from blue, corresponding to 0 m/s, to red, 1 m/s). Ansys EnSight 2023 (Ansys, Inc., Canonsburg, PA) was used to visualize intracardiac flow patterns using streamline representations (TV = Tricuspid Valve; PV = Pulmonary Valve.)

The resulting flow field in the control subject is illustrated in Fig. 4, showing the instantaneous streamline patterns at two representative time points: diastolic filling (left) and systolic ejection (right). Ansys EnSight 2023 (Ansys, Inc., Canonsburg, PA) was used to visualize intracardiac flow patterns using streamline representations. The results demonstrate a high-velocity converging inflow beneath the tricuspid valve (TV), as indicated by elevated velocity magnitudes (red). This tricuspid inflow jet induces the formation of a coherent vortex structure, with rotational flow observed in the subvalvular region. During systole, this rotating blood is redirected and elongated toward the RV outflow tract, consistent with previously reported physiological flow patterns [49–51].

**Fig. 5:**
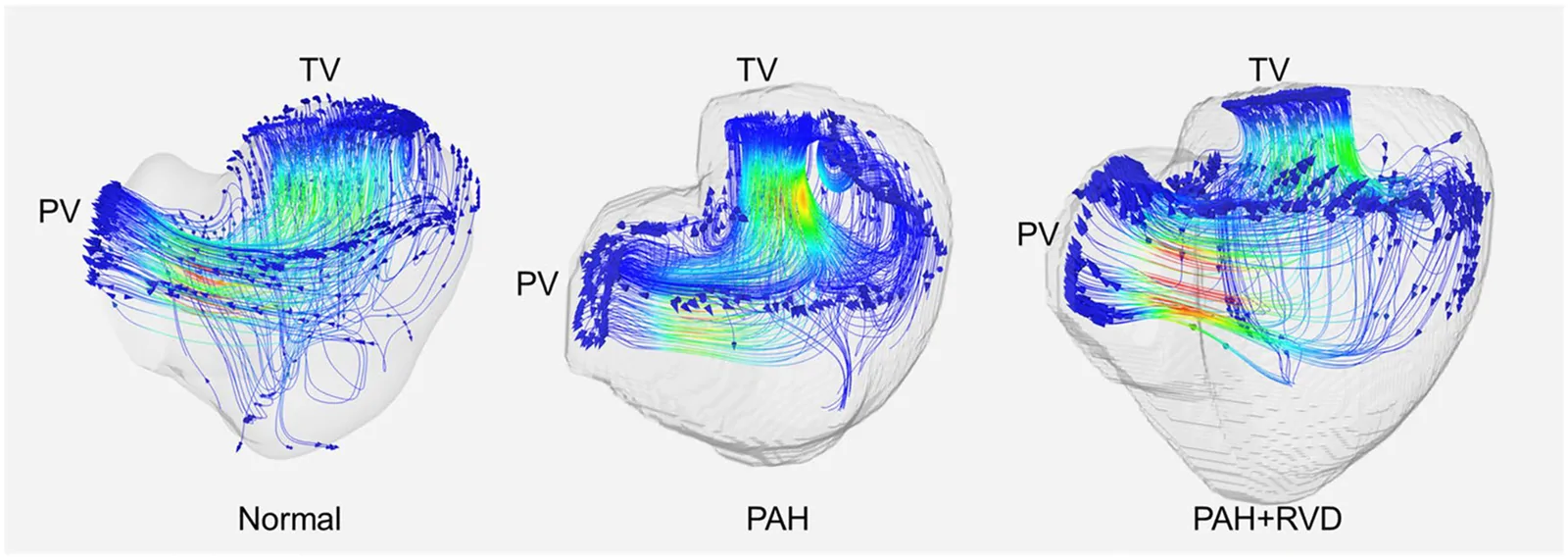
Visualization of the steady-streaming (heartbeat averaged) RV flow field in the three subjects. Flow is visualized in terms of streamlines (emitted from a horizontal plane) and colored by the magnitude of velocity (from blue, corresponding to 0 m/s, to red, 1 m/s). For each simulation, a seeding plane was positioned near the basal region of the right ventricle, parallel to the tricuspid and pulmonary valves, and particle traces were initiated at uniform spatial intervals. These streamline visualizations were generated from the 4D flow fields produced by our computational framework (TV = Tricuspid Valve; PV = Pulmonary Valve.)

Given the inherently complex spatiotemporal evolution of the RV flow field, direct comparison of instantaneous velocity distributions across subjects or cardiac phases can be misleading. To enable qualitative assessment, we examined the steady-streaming flow field, defined as the time-averaged velocity field over an entire cardiac cycle, that captures the net blood motion and the underlying three-dimensional flow organization. For each simulation, a seeding plane was positioned near the basal region of the RV, parallel to the tricuspid and pulmonary valves, and particle traces were initiated at uniform spatial intervals. The steady-streaming patterns for the three representative subjects are presented in Fig. 5. In a control subject (Case #1), streamlines reveal a coherent flow transitioning smoothly from the downward-directed diastolic inflow, curving within the RV body, and converging toward the pulmonary outflow tract. In a PAH patient with preserved RV dimensions (Case #2), the flow remains predominantly confined to the subvalvular region, with reduced penetration toward the RV apex. This effect becomes even more pronounced in cases of PAH with RV dysfunction, as illustrated by a representative subject (Case #3), where the primary vortex forms closer to the tricuspid valve and a substantial apical region demonstrates minimal steady streaming, suggesting increased blood stagnation.

### RV Deformation Analysis

**Fig. 6:**
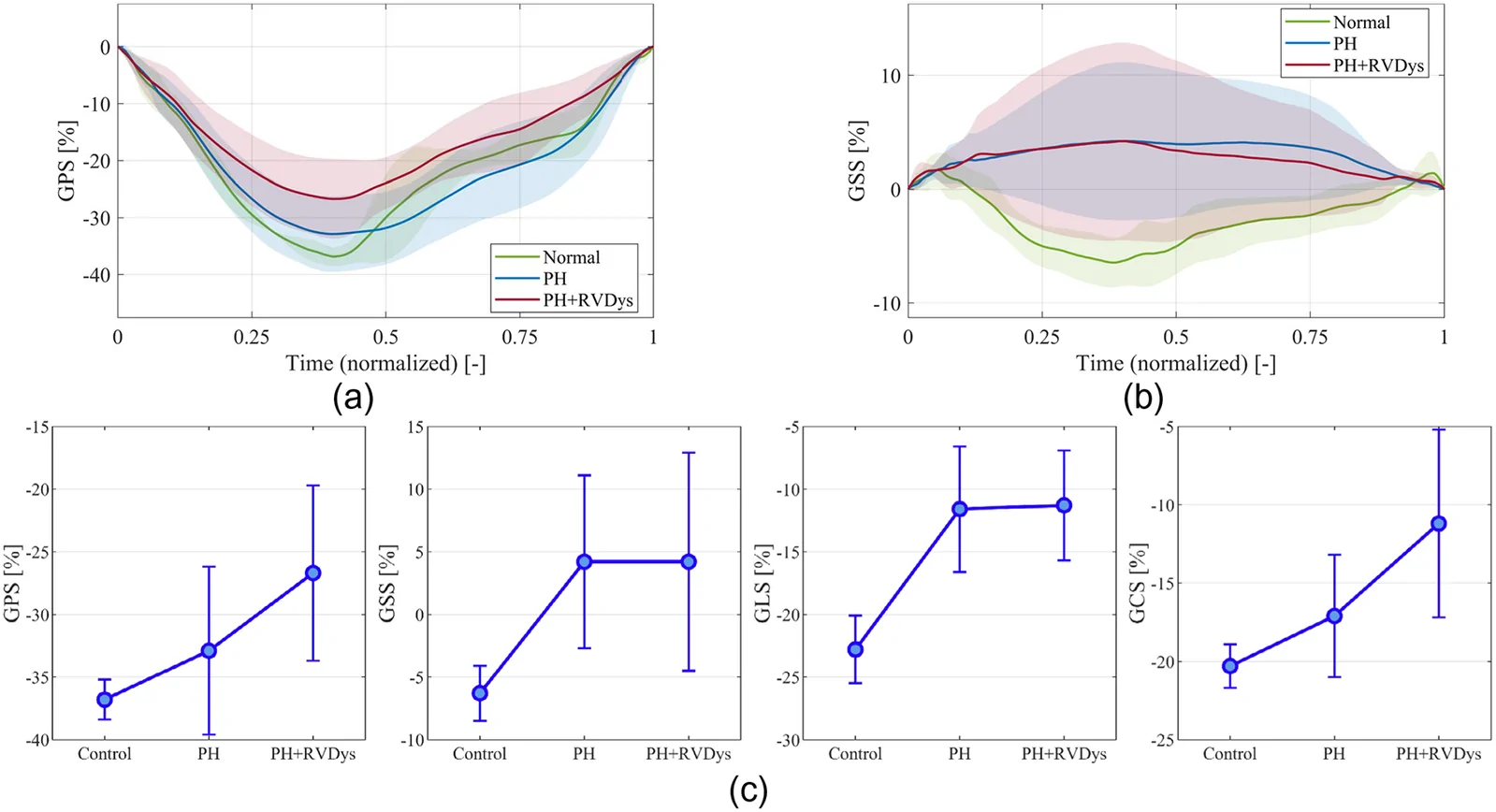
Deformation analysis in the analyzed groups: average time course of a global principal strain (GPS) and **b** global secondary strain (GSS); the surrounding shadow represents the standard deviation, the time period corresponds to one heart cycle from end-diastole (Time = 0), through end-systole (0.4) to next end-diastole (1). Synthetic parameters **c**, (graphic representation of data in Table 2) are reported as average ± standard deviation. The following abbreviations are used: GPS (end-systolic global principal strain), GSS (end-systolic global secondary strain), GLS (end-systolic global longitudinal strain), and GCS (end-systolic global circumferential strain.)

The time course of *GPS* and *GSS* is reported in Fig. 6a and b. To mitigate intersubject variability in heart rate and timing, each cardiac cycle was normalized to a dimensionless interval from 0 to 1, with ED aligned at both 0 and 1, and ES standardized to occur at 0.4. This temporal normalization ensured consistent alignment of systolic and diastolic phases across subjects, enabling more accurate comparison of dynamic strain patterns between groups. *GPS*, which provides an average measure of effective RV deformation, is nearly preserved in PAH and significantly reduced in PAH+RVD. It also presents a somehow continuous profile during diastole with a less marked differentiation of the two filling waves. A more noticeable difference is found in GSS with remarkable evidence of a change of sign that occurs already in PAH and that worsened in PAH+RVD indicating paradoxical systolic lengthening rather than shortening.

The variation of deformation metrics across the groups is reported in Table 2 and shown in Fig. 6c. While *GPS* showed a progressive reduction (in absolute value) across groups, these changes are limited. In contrast, *GSS* exhibited an abrupt and significant shift between control subjects and PAH patients, characterized by a reversal in sign—indicating paradoxical systolic lengthening rather than shortening. This behavior is confirmed in terms of the conventional strain components: *GLS* demonstrated a marked and statistically significant reduction in both PAH and PAH+RVD groups, indicating early and robust impairment of longitudinal shortening; GCS was significantly reduced only in the PAH+RVD group, consistent with the presence of a more advanced disease. This deformation pattern is consistent with septal dyskinesia and mechanical discoordination commonly associated with pressure overload and RV dilatation.

### Hemodynamic Forces in the RV

**Fig. 7:**
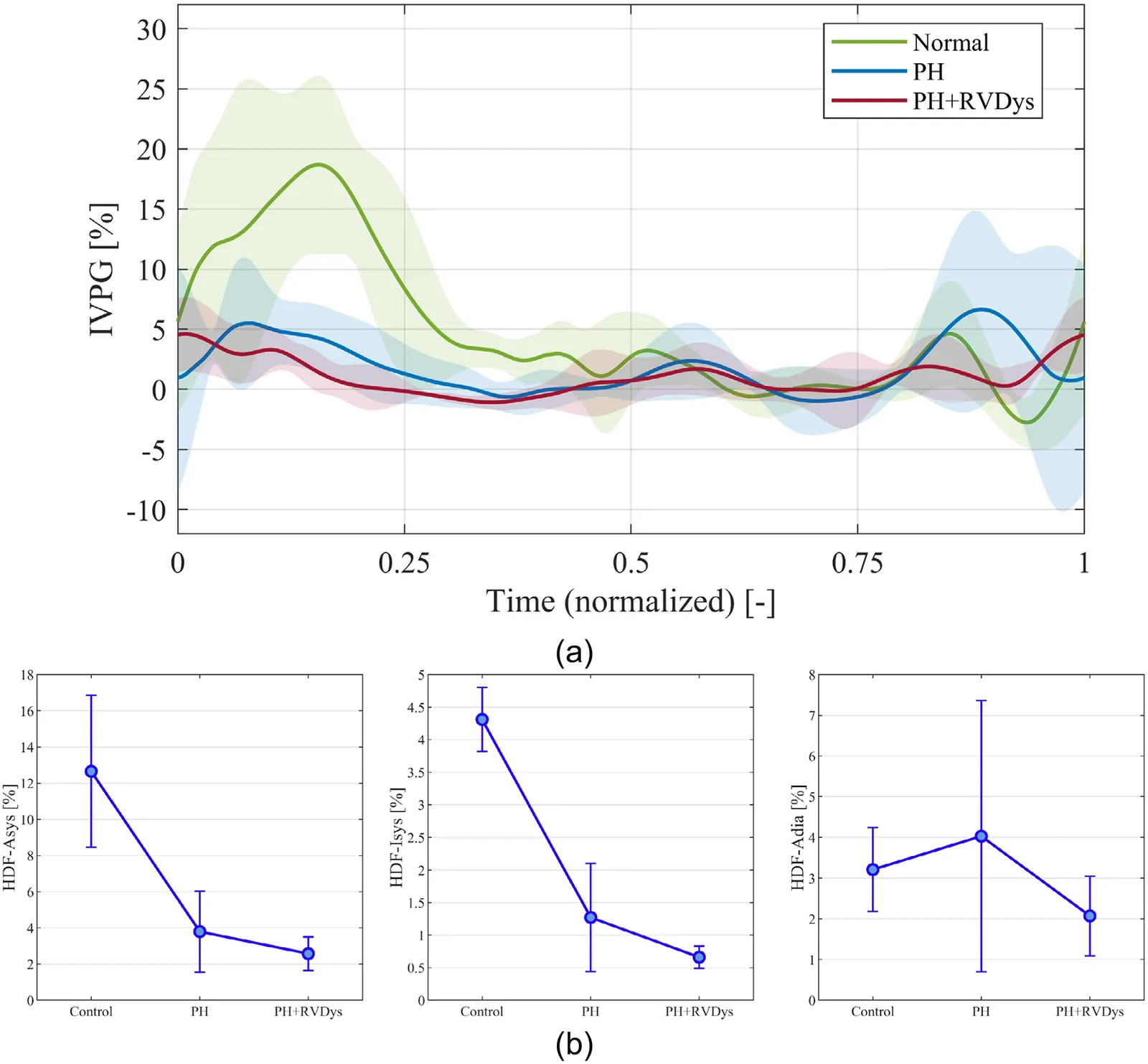
Flow dynamic analysis in the analyzed groups: **a** average time course of the longitudinal hemodynamic force; the surrounding shadow represents the standard deviation, the time period corresponds to one heart cycle from end-diastole (Time = 0), through end-systole (0.4) to next end-diastole (1). Synthetic parameters **b**, (graphic representation of data in Table 2) are reported as average ± standard deviation. The following abbreviations are used: HDF-Asys (Hemodynamic force systolic amplitude), HDF-Isys (Hemodynamic force systolic impulse), HDF-Adia (Hemodynamic force diastolic amplitude)

An initial evaluation of RV fluid dynamic performance was performed through analysis of the longitudinal HDF, whose temporal evolution for the three groups is shown in Fig. 7a, using time-normalized averaging as described previously. Results reveal a marked attenuation of systolic propulsion in both PAH and PAH+RVD groups. This reduction is evident even at mild stages of disease and is highly consistent with the diminished systolic functional markers described previously. In contrast, diastolic HDF patterns remain largely comparable across groups, with no visible shift in timing or amplitude.

Quantitative HDF metrics—including systolic and diastolic amplitudes and the integrated systolic impulse—are presented in Table 2 and visualized in Fig. 7b. These data confirm a substantial reduction in systolic IVPG amplitude, *HDF-Asys*, in both PAH and PAH+RVD relative to controls. Likewise, the systolic impulse, *HDF-Isys*, is significantly diminished in PAH and further reduced in PAH+RVD, highlighting impaired systolic driving forces even in moderately affected patients. In contrast, diastolic IVPG amplitude, *HDF-Adia*, does not differ significantly among groups, indicating relative preservation of diastolic filling pressures despite progressive RV dysfunction.

### RV Flow Energetics

**Fig. 8:**
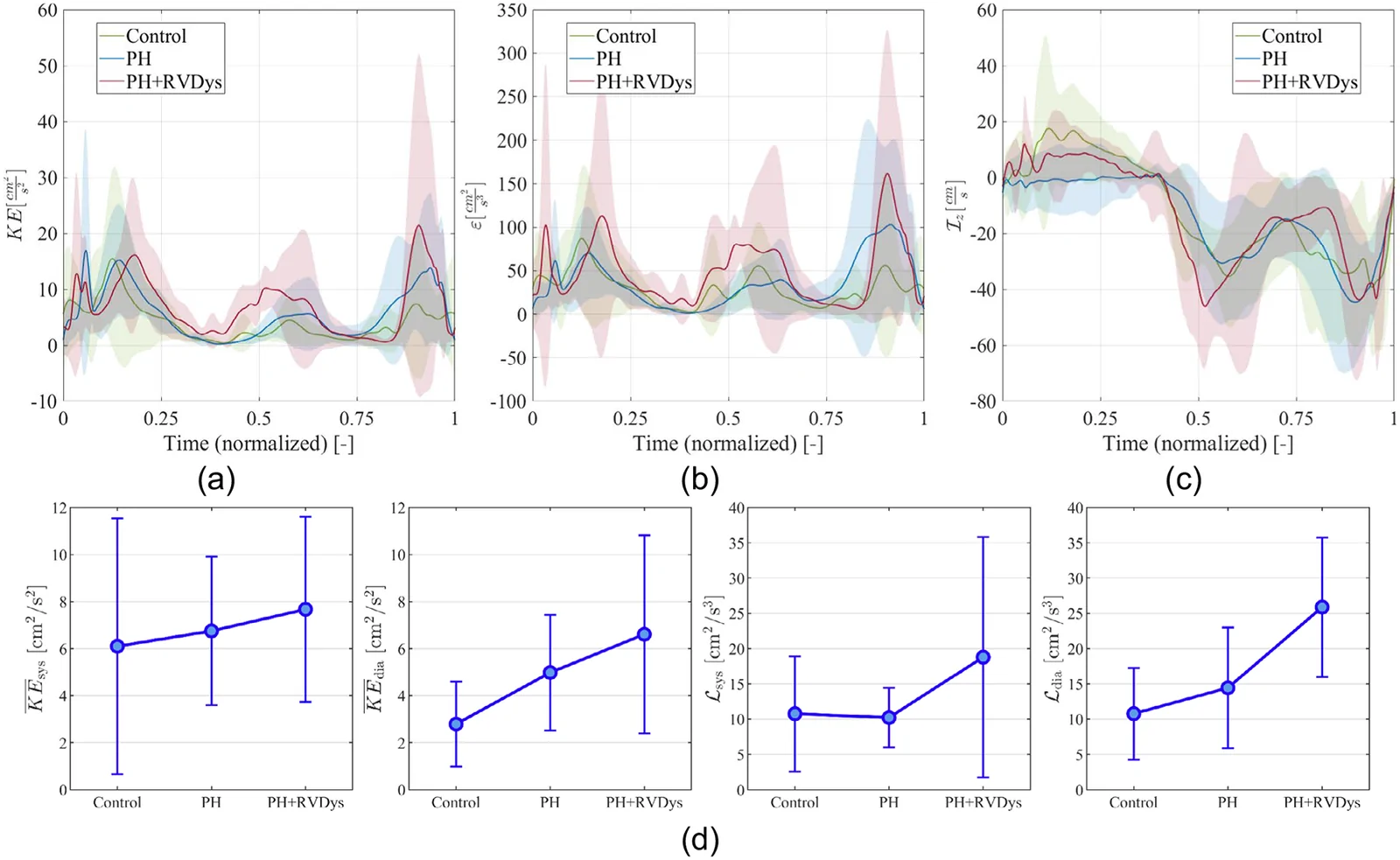
Flow energetic analysis in the analyzed groups: average time course of a normalized kinetic energy (*KE*), **b** rate of energy dissipation (ε), and **c** vertical impulse $\left(I_{z}\right)$; the surrounding shadow represents the standard deviation, the time period corresponds to one heart cycle from end-diastole (Time = 0), through end-systole (0.4) to next end-diastole (1). Synthetic parameters **d**, (graphic representation of data in Table 2) are reported as average ± standard deviation. The following abbreviations are used: ${\bar{\text{KE}}}_{\text{sys}}$ (mean systolic kinetic energy), ${\bar{\text{KE}}}_{\text{dia}}$ (mean diastolic kinetic energy), ${ℒ}_{\text{sys}}$ (systolic energy loss), ${ℒ}_{\text{dia}}$ (diastolic energy loss)

The temporal evolution of energy-based RV flow metrics—mean kinetic energy, *KE*, and vertical diastolic impulse, $I_{z}$,—is shown in Fig. 8a and b, using time-normalized averaging. Although notable intersubject variability is present, the averaged trace profiles exhibit consistent group-related trends, particularly during early and late diastolic filling and a behavior similar to *KE* was found for the rate of energy dissipation, ε. These differences are best quantified in Table 2 and illustrated in Fig. 8c. While no energetic parameters exhibited significant changes during systole, the mean diastolic kinetic energy, ${\bar{\text{KE}}}_{\text{dia}}$, displays an increase in both patients’ groups and a significant increase in diastolic energy loss, ${ℒ}_{\text{dia}}$, was observed in the PAH+RVD group. However, this diastolic energetic change is not accompanied by a change in the diastolic impulse, ${ℐ}_{\text{dia}}$.

Statistical comparison of the time-resolved curves was conducted to evaluate intergroup differences. Outcomes over the normalized cardiac cycle were modeled using Fourier-basis linear mixed-effects models incorporating subject-specific random effects. Following adjustment for multiple comparisons across variables using the Holm correction, constant cycle-wide offsets between groups were not statistically significant (kinetic energy p=0.0747; rate of kinetic energy dissipation p=0.2059; vortex impulse p=0.6695). In contrast, group-specific trajectory differences were highly significant for all variables based on the Group × Phase interaction (all $p<10^{-16}$), indicating that differences between groups vary with cardiac phase rather than reflecting uniform offsets.

## Discussion

### Insights from Deformation Imaging

Growing attention has been given to the clinical significance of cardiac mechanical function, particularly myocardial deformation and strain [31, 52], and, more recently, to intracardiac blood flow dynamics and their interplay with myocardial tissue [53]. Deformation-based metrics have long been recognized as sensitive indicators of subclinical ventricular dysfunction, providing diagnostic value that extends beyond conventional volumetric indexes. Parallel advances in cardiac fluid mechanics have highlighted how flow patterns can influence structural, neuroendocrine, and even epigenetic adaptations similar to those observed during cardiac development [37, 54, 55]. Together, these insights underscore the potential of imaging modalities that integrate myocardial strain with intraventricular flow dynamics to detect early pathophysiological changes before overt clinical deterioration.

In the present study, strain-derived measures captured several key signatures of early and progressive RV dysfunction in PAH. Although global principal strain (GPS) exhibited a minor progressive decline with disease severity, longitudinal strain (GLS) was significantly reduced even in PAH patients without overt RV dilation (Table 2, p=0.01), indicating its sensitivity as an early marker of impaired contractility. Global circumferential strain (GCS) exhibited significant further deterioration only in patients with established RV dysfunction, consistent with progressive geometric and mechanical remodeling. Of particular interest is the pattern of secondary strain (GSS) that revealed a striking sign reversal in PAH, becoming persistently positive throughout systole. This paradoxical systolic lengthening reflects septal dyskinesia and mechanical discoordination, aligning with established concepts of RV–LV uncoupling under chronic pressure overload.

Temporal profiles of GPS and GSS further highlighted the progressive loss of coordinated deformation, with attenuation of early and late diastolic relaxation waves in PAH. These changes collectively illustrate how RV mechanical remodeling manifests not only as reduced contractile amplitude but also as disrupted spatial coordination of myocardial deformation.

### Advancements in Echocardiographic Flow Imaging

While myocardial strain imaging is clinically well established, the translation of intracardiac flow assessment into routine practice remains limited. Techniques such as phasecontrast MRI (4D Flow MRI) are considered reference standards [56–58] but are constrained by high cost, extended acquisition and processing times, and limited availability. Echocardiographic techniques—including color Doppler [59, 60] and 2D Echo-PIV [27, 44, 61]—offer greater accessibility but fail to capture the complex three-dimensional flow structures of the right ventricle [49, 50, 62–64]. Thus, the clinical relevance of flow imaging depends less on high-resolution, voxel-wise velocity fields and more on extracting clinically meaningful flow-derived markers that reflect global hemodynamic performance.

The present work advances the extraction of clinically meaningful, flow-derived markers of global hemodynamic performance through two complementary techniques. First, a theoretical framework [38] enables estimation of the hemodynamic force exchanged between blood and myocardium, which, once normalized by the RV volume, represents the average value of intraventricular pressure gradient—a fundamental determinant of ventricular function [37]. In addition, V-Echo-PIV, as a volumetric extension of Echo-PIV, integrates RV boundary motion with mass conservation of blood flow, enabling the extraction of coherent 3D flow patterns from standard contrast echocardiography. V-Echo-PIV enables the tracking of contrast microbubbles in three-dimensional space throughout their swirling trajectories, overcoming a key limitation of 2D Echo-PIV, where bubbles quickly move out of the imaging plane. However, implementing V-Echo-PIV using ultrasound data acquired with a matrix-array probe is currently constrained by the limited temporal resolution of conventional clinical 3D echocardiography systems. As high-frame-rate ultrasound technologies mature [65, 66], the precision and diagnostic range of volumetric flow imaging will likely expand.

The present implementation of V-Echo-PIV demonstrates the ability to extract meaningful RV flow dynamics using standard clinical echocardiography systems. The primary aim of this study was methodological validation and feasibility of advanced flow imaging techniques; therefore, the clinical findings should be considered preliminary, as they are derived from a limited population. Moving forward, this approach should be supported by systematic validation before potential translation into routine clinical practice.

Given the absence of an absolute ground truth, validation must carefully account for technical differences across imaging modalities. More importantly, it should not focus on achieving instantaneous, pointwise accuracy of velocity fields, but rather on assessing the method’s ability to reliably estimate clinically relevant parameters. In this context, the proposed approach emphasizes the reconstruction of physiologically meaningful flow patterns and the estimation of global energetic metrics, which may provide valuable insight into early diagnosis and longitudinal monitoring of RV dysfunction.

### Integrating Myocardial Mechanics and Flow Dynamics for Monitoring PAH Progression

This study demonstrates the feasibility of integrating 3D echocardiographic measures of RV mechanics with advanced flow-derived metrics to characterize the hemodynamic consequences of PAH. Across disease stages, we observed distinct changes in intraventricular pressure gradients and energetics that complement conventional volumetric and strain-based findings.

The analysis of HDF revealed a visible, drastic reduction in systolic propulsion, with both systolic amplitude and systolic impulse significantly decreased in PAH and PAH+RVD patients. Notably, these reductions were present even in patients without major RV enlargement, suggesting that systolic HDF may serve as an early indicator of compromised contractile force transmission. By contrast, diastolic HDF amplitude remained preserved, suggesting maintained filling mechanics despite impaired systolic performance. In contrast, both mean kinetic energy and energy loss display more pronounced diastolic variations across groups. Increased energy dissipation was observed in all PAH patients and is particularly notable given normalization by RV volume, suggesting impaired intraventricular energy transfer efficiency in PAH. The vortex impulse remains relatively unchanged, suggesting that although the process of vortex formation is preserved, its energetic contribution to RV flow differs between normal and pathological states. These results highlight the complementary diagnostic value of integrating blood–tissue dynamic interactions with energetic flow analyses for characterizing RV dysfunction in PAH.

The combination of myocardial deformation, forces, and flow energetic analyses offers a physiologically comprehensive perspective on early RV changes in PAH. GLS and GSS emerged as sensitive markers of early mechanical impairment, while systolic HDF provided a robust hemodynamic correlate of reduced contractile force transfer. Energetic abnormalities, particularly elevated diastolic energy loss, appeared predominantly in patients with more advanced RV dysfunction, supporting their potential role in disease staging rather than early detection.

While these findings suggest that integrated fluid mechanics metrics can reliably differentiate healthy controls from patients across the spectrum of PAH severity, larger studies with clinical outcomes are needed to validate their prognostic and diagnostic utility. Ultimately, the fusion of deformation imaging with RV flow dynamics may contribute to improved early detection, risk stratification, and monitoring of right-heart dysfunction in pulmonary hypertension.

### Limitations and Future Directions

This study has limitations that should be acknowledged. It was based on a relatively small, carefully selected cohort and was primarily designed to demonstrate the technical feasibility of a noninvasive framework for assessing PAH. The small sample size (n=5 per group) limits statistical power; therefore, the study should be regarded as exploratory and hypothesis-generating rather than definitive. Larger, adequately powered studies are on the way to evaluate reproducibility and define the diagnostic performance of the proposed indices. In this respect, the accuracy and reproducibility of V-Echo-PIV are still to be confirmed in a systematic comparative study. Despite these limitations, the findings suggest that subtle changes in RV flow and mechanics are detectable early in PAH, supporting the potential value of this integrated approach for future screening and monitoring applications.

In conclusion, this study demonstrates the feasibility of a noninvasive framework for evaluating RV function in pulmonary arterial hypertension by integrating intracavitary flow dynamics with myocardial deformation analysis using three-dimensional echocardiography. The approach reveals stage-dependent hemodynamic abnormalities, including reduced systolic propulsion evident in early disease and markedly increased diastolic energy loss in advanced RV dysfunction, highlighting their potential diagnostic value. These flow-derived findings complement the deterioration in myocardial mechanics identified by deformation analysis, characterized by significant reductions in longitudinal strain and early reversal of secondary strain. Collectively, the combined assessment of RV deformation, intraventricular pressure gradients, and energetic metrics captures a broad spectrum of processes associated with progressive RV dysfunction in PAH.

While validation in larger cohorts is warranted, this physiologically grounded framework offers a noninvasive strategy with potential to enhance early detection, disease staging, and longitudinal monitoring in PAH.

## Figures and Tables

**Table 1:** Main demographic clinical and volumetric parameters for the three study groups—Control, PAH (pulmonary arterial hypertension), and PAH+RVD (pulmonary arterial hypertension with right ventricular dysfunction or RV enlargement)—are presented as mean ± standard deviation where appropriate

	Control (n=5)	PAH (n=5)	p	PAH+RVD (n=5)	p	$p_{\text{KW}}$
Age [years]	42.4 ± 16.1	54.2 ± 21.8	0.57	57.6 ± 16.0	0.87	0.38
Gender [F + M]	2 + 3	4 + 1	0.50	3 + 2	0.70	0.46
RVSP [mmHg]	23.3 ± 5.8	61.7 ± 27.6	0.10	63.0 ± 15.9	0.10	0.07
TAPSE [cm]	2.4 ± 0.2	1.67 ± 0.12	0.03	1.6 ± 0.44	0.08	0.06
RVEDV [ml]	162.1 ± 74.9	190.6 ± 56.6	0.69	230.8 ± 35.0	0.22	0.36
RVESV [ml]	73.0 ± 39.7	115.8 ± 41.9	0.22	151.9 ± 31.9	0.02	0.04
RVEF [%]	56.3 ± 4.5	40.0 ± 7.8	0.02	34.0 ± 11.0	0.01	0.01

*RVSP* right ventricular systolic pressure, *TAPSE* tricuspid annular plane systolic excursion, *RVEDV* right ventricular end-diastolic volume, *RVESV* right ventricular end-systolic volume, and *RVEF* right ventricular ejection fraction

p-values from Mann-Whitney U tests, comparing each patient group with the Control group, are provided, and p-values from omnibus Kruskal–Wallis test $\left(p_{\text{KW}}\right)$

**Table 2:** Deformation parameters for the three study groups—Control, PAH (pulmonary arterial hypertension), and PAH+RVD (pulmonary arterial hypertension with right ventricular dysfunction or enlargement)—are reported as mean ± standard deviation

	Control (n=5)	PAH (n=5)	p	PAH+RVD (n=5)	p	$p_{\text{KW}}$
GPS [%]	−36.8 ± 1.6	−32.9 ± 6.7	0.69	−26.7 ± 7.0	0.06	0.10
GSS [%]	−6.3 ± 2.2	4.2 ± 6.9	0.02	4.2 ± 8.7	0.15	0.07
GLS [%]	−22.8 ± 2.7	−11.6 ± 5.0	0.01	−11.3 ± 4.4	0.01	0.01
GCS [%]	−20.3 ± 1.4	−17.1 ± 3.9	0.15	−11.2 ± 6.0	0.01	0.03
HDF-Asys [%]	12.65 ± 4.20	3.79 ± 2.24	0.01	2.57 ± 0.93	0.01	0.02
HDF-Isys [%]	4.31 ± 0.49	1.27 ± 0.83	0.01	0.66 ± 0.17	0.01	0.00
HDF-Adia [%]	3.21 ± 1.03	4.03 ± 3.33	0.84	2.07 ± 0.98	0.15	0.40
${\bar{KE}}_{\text{sys}}\left[{\text{cm}}^{2}/{\text{s}}^{2}\right]$	6.10 ± 5.44	6.75 ± 3.16	0.42	7.67 ± 3.94	0.42	0.51
${\bar{KE}}_{\text{dia}}\left[{\text{cm}}^{2}/{\text{s}}^{2}\right]$	2.79 ± 1.80	4.98 ± 2.46	0.06	6.61 ± 4.21	0.06	0.05
${ℒ}_{\text{sys}}\left[{\text{cm}}^{2}/{\text{s}}^{2}\right]$	10.76 ± 8.17	10.22 ± 4.22	0.69	18.79 ± 17.03	0.69	0.78
${ℒ}_{\text{dia}}\left[{\text{cm}}^{2}/{\text{s}}^{2}\right]$	10.77 ± 6.51	14.43 ± 8.55	0.31	25.88 ± 9.88	0.03	0.07
${ℐ}_{\text{dia}}[\text{cm}/\text{s}]$	−25.00 ± 4.94	−23.87 ± 10.15	0.84	−24.54 ± 11.65	0.84	0.93

*GPS* end-systolic global principal strain, *GSS* end-systolic global secondary strain, *GLS* end-systolic global longitudinal strain, and *GCS* end-systolic global circumferential strain, *HDF-Asys* Hemodynamic force systolic amplitude, *HDF-Isys* Hemodynamic force systolic impulse, *HDF-Adia* Hemodynamic force diastolic amplitude, ${\bar{KE}}_{\text{sys}}$ mean systolic kinetic energy, ${\bar{KE}}_{\text{dia}}$ mean diastolic kinetic energy, ${ℒ}_{\text{sys}}$ systolic energy loss, ${ℒ}_{\text{dia}}$ diastolic energy loss, and ${ℐ}_{\text{dia}}$ mean diastolic impulse

p-values from Mann-Whitney U tests, comparing each patient group with the Control group, are provided, and p-values from omnibus Kruskal–Wallis test $\left(p_{\text{KW}}\right)$

## Data Availability

The data that support the findings of this study are available from the corresponding author upon reasonable request.
